# Empyema

**Published:** 1893-02-18

**Authors:** 


### The Hospital Clinic.

[*The Editor will be glad to receive offers of co-operation and contributions from members of the profession. All letters should be addressed to* The Editor, The Lodge, Porchester Square, London, W.]

### ST. THOMAS'S HOSPITAL.

### Empyema.

In opening up the question of the treatment of empyema, our first thought is that it is often but making the best of a bad business—stepping in to patch up a "case" what would have been so much better prevented; for we feel that with more careful observation on the part of those by whom the cases brought to hospital with an empyema have been first attended, many a simple pleuritic effusion could have been nipped in the bud, and even, if severe, cured before suppuration by timely use of the aspirator. It seems certain that in many, if not most such cases, it is the neglect of early use of the aspirator, rather than its misuse, which is responsible for a serous effusion becoming purulent.

However, in addition to the above cases, there are those in which the medical attendant is never called in until the suppuration has occurred, and others arising from the spontaneous rupture of a purulent focus in the lungs, or neighbouring abdominal viscera, or of an abscess connected with tubercular disease of the spinal column, ribs, or sternum, and, lastly, the rarer traumatic cases; for all these the treatment is within certain limits the same, and we propose in the following article to give a short sketch of the practice generally followed in St. Thomas's Hospital.

Cases of empyema, usually in the first instance, come under treatment on the medical side, and the aspirator or exploratory syringe is generally used, chiefly for diagnostic purposes. In a few cases, however, the simple withdrawal of the pus in this way has been followed by so much improvement that before surgical interference has been invited aspiration has been repeated once or twice upon reaccumulation of the fluid, and though we believe drainage in the ordinary way has eventually been necessary in all but a very few cases, still the delay has been beneficial in allowing time for feeding up the patient, and it is probable that, were the cases seen sufficiently early, the efficiency of simple aspiration as a possible means of cure would be proved—certainly there are cases on record of purulent collections in other serous cavities, notably the knee-joint, which have been cured by simple aspiration.

In all ordinary cases the routine treatment has been incision, subperiosteal resection of a small piece of rib, and drainage by means of a double piece of drainage tube.

The operation is in almost every case performed under anæsthesia, ether or chloroform being used according as seems best suited to the age of the patient and the condition of the heart or lungs. After the chest wall has been carefully washed and sponged with antiseptic solution, the site of the incision has been selected according to the size and situation of the empyema; where the collection of pus is small and localised, the incision is made over the centre of the collection; where it is more extensive, the point most frequently chosen has been near the posterior axillary line, just in front of the edge of the latissimus dorsi, or, at any rate, between this and the mid-axillary line. Cutting straight down on to the rib (generally the sixth), midway between the upper and lower borders, through the periosteum, the bone is bared with the elevator for about one inch. The piece bared is then resected with bone forceps, the parietal pleura and the intra-costal tissues being protected by a flat metal retractor passed between them and the rib.

The parietal pleura is then carfully punctured with a blunt director at a point corresponding to the middle of the rib to avoid a wound of the inter-costal vessels, and on the appearance of pus the puncture is dilated up first with the forceps, after Hilton's method, and then with the finger. After the pus has been allowed to escape, and any caseous curds clinging about the wound have been removed and all hemorrhage arrested by forceps and ligature, a large double drainage tube is inserted, one portion of it long enough to reach to the end of the cavity, the ends being secured from risk of slipping in by safety pins passed through them.

A copious dressing of antiseptic gauze and absorbent wool is then applied and the patient put back to bed. Fresh layers of wool should be packed on should the discharge come through the first dressing, and unless there are signs of hemorrhage going on the patient should not be disturbed for twenty-four hours. Then the whole dressing should be renewed, and after this changed occasionally at intervals of one, two, or more days, depending upon the amount and quality of the discharge. The tube is gradually shortened as the cavity diminishes in size, but it is never safe to remove it altogether while there is any length of cavity inside for fear of reaccumulation of the pus.

In long standing cases, particularly in adults, where the expansion of the lung is inadequate, and the chest wall too rigid to fall in, Estlander's operation of resection of portions of several adjacent ribs over the site of the cavity has been employed with success. Thoracoplasty, or the operation of dividing the ribs over the empyema at two points to allow the chest wall to fall in and obliterate the cavity has not, we understand, been practised.

It is always advisable that operations for empyema should be performed as rapidly as possible, owing to the difficulty of respiration and consequent danger of syncope, besides the risks arising from the effect of the anæsthetics on lungs, almost always themselves diseased. The question as to whether the empyema cavity should be washed out is still debated, and certainly the practice must be held to be dangerous, unless the cavity is a small one, and the lotion warmed up to about 100 deg. F., and then only allowed to run in very gently in small quantities at a time; boracic lotion or sterilised water, warmed to a temperature of about 100° F., being probably the best fluids to use.

One small point in conclusion is worth mention, namely, that in a few cases collections of pus occur outside the pleura, but otherwise exactly resembling empyemata. In operating on such cases, unless care is taken when an incision is made to notice exactly where the first pus is found, the pleura may be opened up unnecessarily under the idea that the pus seen is really escaping from an accidental opening of the pleura, made during the detachment of the periosteum from the rib. The after treatment calls for no special notice. Beyond supporting the strength, changing the dressing when necessary, and shortening the tube so that it is constantly kept a little shorter than the length of the empyema cavity, there is nothing to be done so long as the cavity is steadily diminishing and the lung expanding. Should this be arrested, Estlander's or some allied operation may be called for, or even repeated, if not successful at first.

